# EHMTI-0130. Questioning the presence of iron deficiency anemia in migrane and tension type headache: a case control study

**DOI:** 10.1186/1129-2377-15-S1-D25

**Published:** 2014-09-18

**Authors:** S Gur Ozmen, R Karahan Ozcan

**Affiliations:** 1 Neurology, Igdir State Hospital, Igdir, Turkey; 2 Neurology, Gebze Fatih State Hospital, Kocaeli, Turkey

## Background

Generally speaking headache is an accepted symptom of iron deficiency anemia (IDA). However, it is not very clear which type of headache has an association with IDA, either migrane or tension type headache.

## Aim

To evaluate the presence of iron deficiency anemia (IDA) in migraine and TTH patients, as well as in controls.

## Method

A case-control clinical study was conducted to investigate the presence of IDA in 169 migraine, 165 TTH patients and in 170 healthy controls. Migraine and TTH diagnosis was settled according to the International Classification of Headache Disorders-II diagnostic criteria, and each subject was evaluated in terms of headache characteristics including severity, frequency, duration of the headache attack, and the duration of the disease. Routine hematological analysis was applied for all of the groups. Descriptive statistics and multivariate regression analysis were performed.

## Results

IDA was observed in 21% of the patients with migraine and in 15% with TTH whereas it was observed in 12% of the healthy controls. IDA was found to be significantly higher in patients with migraine than controls (P = 0.03). IDA was independently associated with migraine in multivariate model (OR: 1.88; CI 95% 1.01-3.50, P = 0.04). Age (P = 0.02) and duration of menstrual bleeding (P = 0.006) were the factors affecting presence of IDA in migraine. There was no association between TTH and IDA.

## Conclusion

Our results suggest that IDA is prevalent in migraine patients but not in TTH. This finding should be validated in further patient series.

No conflict of interest.

